# Integrated Analysis of the lncRNA-miRNA-mRNA ceRNA Network in the Spleen of *Acrossocheilus fasciatus* Infected with *Citrobacter freundii*

**DOI:** 10.3390/biology15211994

**Published:** 2026-11-04

**Authors:** Wenbin Fang, Renxuan Huang, Qing Fang, Rongquan Zheng, Shanjian Zheng

**Affiliations:** 1 College of Life Sciences, Zhejiang Normal University, Jinhua 321004, China; 202420501007@zjnu.edu.cn (W.F.); zhengrq@zjnu.cn (R.Z.); 2 Key Lab of Wildlife Biotechnology and Conservation and Utilization of Zhejiang Province, Zhejiang Normal University, Jinhua 321004, China

**Keywords:** *Acrossocheilus fasciatus*, *Citrobacter freundii*, whole transcriptome, LncRNA, ceRNA

## Abstract

*Acrossocheilus fasciatus* is an economically important freshwater fish species in China, and infection with *Citrobacter freundii* poses a serious threat to the sustainable development of its aquaculture industry. In this study, histopathological examination, TUNEL staining, immune physiological assays, and whole-transcriptome sequencing were integrated to systematically characterize the expression profiles of lncRNAs, circRNAs, miRNAs, and mRNAs in the spleen of *A. fasciatus* following *C. freundii* infection. The results showed that infection caused marked tissue damage in the intestine, spleen, and kidney, significantly increased splenic cell apoptosis, and induced pronounced changes in immune physiological parameters. Whole-transcriptome analysis identified 88 differentially expressed lncRNAs, 193 differentially expressed circRNAs, 140 differentially expressed miRNAs, and 1935 differentially expressed mRNAs. KEGG enrichment analysis revealed that the differentially expressed mRNAs were mainly enriched in immune-related pathways, including neutrophil extracellular trap formation, complement and coagulation cascades, antigen processing and presentation, cytokine–cytokine receptor interaction, phagosome, chemokine signaling pathway, and IL-17 signaling pathway. Furthermore, a bioinformatically predicted lncRNA-miRNA-mRNA ceRNA network was constructed, comprising 10 mRNAs, 14 lncRNAs, and 22 miRNAs, which formed 44 putative regulatory interactions. In this network, *miR-212-y* may mediate the regulation of *Il12rb2* and *PIK3R1*, whereas *miR-8-y* may be involved in the regulation of *Ccr9* and *PIK3R1*. The qRT-PCR validation results were consistent with the RNA-seq expression trends. This study provides the first comprehensive characterization of non-coding RNA and mRNA expression profiles in the spleen of *A. fasciatus* after *C. freundii* infection and identifies potential ceRNA regulatory networks, providing a valuable reference for elucidating antibacterial immune mechanisms and screening candidate molecular markers for disease resistance research in *A. fasciatus*.

## Simple Summary

*Acrossocheilus fasciatus* is an economically valuable freshwater fish species widely farmed in southern China. In recent years, infections caused by the bacterium *Citrobacter freundii* have led to widespread disease and mortality in farmed stocks, posing a serious threat to the sustainable development of the local aquaculture industry. This study aims to investigate how this fish species responds to bacterial infection at both the tissue and molecular levels. We examined tissue damage in the intestine, spleen and kidney after infection, tracked changes in immune indicators, and analyzed expression alterations of various genes and non-protein-coding RNA molecules in the spleen. We built a gene regulatory network and identified several key molecules potentially involved in antibacterial immune responses. These findings advance our understanding of fish antibacterial defense mechanisms and provide a scientific foundation for breeding disease-resistant fish strains and supporting healthy aquaculture development.

## 1. Introduction

With the continued expansion and intensification of freshwater aquaculture, bacterial diseases have become one of the major constraints on fish health management and the sustainable development of the aquaculture industry. High stocking densities, fluctuations in water quality, unconsumed feed, and chronic stress in aquaculture environments can disrupt mucosal barrier function and immune homeostasis in fish, thereby increasing their susceptibility to opportunistic bacterial infections. *Citrobacter freundii*, a Gram-negative bacterium belonging to the genus *Citrobacter* within the family Enterobacteriaceae, is an opportunistic pathogen with zoonotic potential in humans, livestock, and fish [1,2]. To date, *C. freundii* has been identified in several cultured fish species, including crucian carp (*Carassius auratus*), grass carp (*Ctenopharyngodon idellus*), and largemouth bass (*Micropterus salmoides*) [3,4,5].

*Acrossocheilus fasciatus*, commonly known as the “freshwater grouper,” is an emerging economically important freshwater fish species in southern China. It is mainly distributed in mountain streams in Zhejiang, Jiangxi, Taiwan, and other regions of China [6,7], and has considerable aquaculture value as well as ecological research significance. In recent years, with the expansion of artificial breeding and farming practices, *C. freundii* infection has become a serious obstacle to the healthy development of *A. fasciatus* aquaculture. Therefore, there is an urgent need to develop *A. fasciatus* strains with enhanced disease resistance.

Transcriptome sequencing enables genome-wide profiling of gene expression changes before and after infection and facilitates the identification of immune-related pathways, key regulatory factors, and potential molecular markers associated with disease resistance from large sets of differentially expressed genes. It has therefore become one of the core technologies for elucidating the links among pathogen infection, host immune responses, tissue damage, and antibacterial defense mechanisms in aquatic disease research [8,9,10,11]. In the head kidney and spleen of Chinese sturgeon (*Acipenser sinensis*) infected with *C. freundii*, numerous immune-related genes were significantly dysregulated [12]. In infected zebrafish (*Danio rerio*), differentially expressed genes were mainly involved in immune processes such as complement activation, antigen processing and presentation, coagulation, autophagy, and apoptosis [13]. A recent transcriptomic study has characterized the mRNA-level splenic immune response of *A. fasciatus* against *C. freundii* infection, but the post-transcriptional regulatory network mediated by non-coding RNAs remains largely unexplored [14]. However, because fish immune responses are highly tissue-specific and infection-stage-dependent, mRNA-based analyses alone are insufficient to fully reveal the complex post-transcriptional regulatory mechanisms underlying the infection process [15].

Non-coding RNAs (ncRNAs) are a class of RNA molecules that lack protein-coding potential [16]. Initially, ncRNAs were regarded as transcriptional noise [17]. With the rapid development of high-throughput sequencing technologies, increasing evidence has demonstrated that ncRNAs are important regulators of gene expression [18,19]. They are widely involved in multiple cellular biological processes, including precursor RNA processing, intron splicing, genome remodeling, cell development and differentiation, and epigenetic regulation [20,21,22,23].

These ncRNAs are generally classified into three major types: microRNAs (miRNAs), long non-coding RNAs (lncRNAs), and circular RNAs (circRNAs). MiRNAs are typically 21–24 nucleotides in length. They can bind to the 3′ untranslated region, 5′ untranslated region, or coding sequence of target mRNAs, thereby repressing translation or promoting mRNA degradation. In teleost fish, miRNAs have been shown to regulate viral replication during infection by targeting host genes involved in interferon and apoptosis-related signaling pathways [24,25,26,27]. They have also been widely implicated in the regulation of host immune responses during pathogen infection in fish [28,29,30]. By contrast, lncRNAs are ncRNAs longer than 200 nucleotides [31] and can directly affect target gene expression through cis- and trans-regulatory mechanisms [32]. Accumulating evidence indicates that lncRNAs participate in various biological and physiological processes, including metabolism, cell proliferation, differentiation, migration and apoptosis [33,34]. Circular RNAs (circRNAs) are a class of ncRNAs characterized by covalently closed-loop structures generated through the back-splicing of precursor mRNAs, in which the 3′ and 5′ ends are joined by a splice junction [35]. The competitive endogenous RNA (ceRNA) network has emerged as an important regulatory model. According to this model, lncRNAs can act as ceRNAs or miRNA sponges by competing for shared miRNA binding sites, thereby reducing miRNA-mediated repression of target mRNAs and forming a dynamic, multilayered regulatory network [36,37]. CeRNA regulatory networks have been characterized in multiple aquatic organisms, including yellow catfish (*Pelteobagrus fulvidraco*), leopard coral grouper (*Plectropomus leopardus*), and oysters [38,39,40].

Although previous studies have investigated immune responses in fish infected with *C. freundii* from the perspective of mRNAs or specific ncRNAs, a systematic integrative analysis of lncRNAs, circRNAs, miRNAs, and mRNAs in *A. fasciatus* following *C. freundii* infection remains limited. In this study, the pathogenic effects of *C. freundii* on *A. fasciatus* were evaluated using histopathological observation, apoptosis detection, enzyme activity assays, and immune parameter measurements. Meanwhile, whole-transcriptome sequencing was performed to comprehensively characterize transcriptomic changes in *A. fasciatus* before and after *C. freundii* infection. Differentially expressed lncRNAs (DElncRNAs) and circRNAs (DEcircRNAs) induced by *C. freundii* infection were identified, and a lncRNA–miRNA–mRNA ceRNA regulatory network was constructed. To our knowledge, this study provides the first ceRNA regulatory network associated with *C. freundii* infection in *A. fasciatus*, offering new insights into fish immune regulation. The identified ceRNA network may also provide potential molecular markers for improving disease resistance in *A. fasciatus*.

## 2. Materials and Methods

### 2.1. Sample Collection and Bacterial Infection

The *C. freundii* strain used in this study was previously isolated from diseased *A. fasciatus* in our laboratory. The strain was inoculated into brain heart infusion (BHI) broth and cultured overnight in a shaking incubator at 28 °C and 220 rpm.

A total of 360 healthy *A. fasciatus*, approximately 6 months old, were obtained from Quzhou, Zhejiang Province, China. Prior to the experiment, all fish were acclimated in an indoor recirculating aquaculture system for 14 days. Subsequently, 180 fish were randomly divided into six groups, with 30 fish in each group. Five groups were used for bacterial challenge, and one group served as the control. Fish in the challenge groups were intraperitoneally injected with 0.1 mL of *C. freundii* suspension at concentrations of 1 × 10^8^, 1 × 10^7^, 1 × 10^6^, 1 × 10^5^, and 1 × 10^4^ CFU/mL, respectively. Fish in the control group were injected with an equal volume of sterile 1 × PBS. After injection, all fish were maintained in tanks without water recirculation. Mortality was continuously monitored and recorded for 72 h (Figure S1). Based on the mortality data, the median lethal dose (LD_50_) was calculated using Probit regression analysis and used to establish the subsequent infection model [41]. The LD_50_ was determined to be 2.08 × 10^6^ CFU/mL (equivalent to 2.08 × 10^5^ CFU per fish).

The remaining 180 *A. fasciatus* were used to establish the formal infection model. Fish were randomly assigned to six independent tanks (30 fish per tank), with three tanks as the infected group and three tanks as the control group. Each tank was treated as an independent experimental unit to avoid pseudoreplication. Fish in the infected tanks were intraperitoneally injected with 0.1 mL of bacterial suspension at 2.08 × 10^6^ CFU/mL (the LD_50_ dose), whereas fish in the control tanks were injected with an equal volume of sterile 1 × PBS. After injection, all fish were maintained under the same water conditions. All experimental procedures in this study were approved by the Experimental Animal Ethics Committee of Zhejiang Normal University and were conducted in accordance with relevant guidelines (No.: ZSDW2025076).

Before sampling, all fish were anesthetized with MS-222 at a final concentration of 60 mg/L. Tissue sampling was performed at 0, 24, 48, and 72 h post-infection (hpi). At each time point, 6 fish were randomly selected from each tank for tissue collection. Spleen samples collected across all four time points were used for antioxidant enzyme activity assays and immune-related protein quantification. Based on pre-experimental results showing prominent splenic tissue injury and fully activated innate and humoral immune responses at 72 hpi, spleen samples from this time point were selected for whole-transcriptome sequencing and subsequent qRT-PCR validation. For sequencing and qRT-PCR analysis, spleen tissues from 3 randomly selected fish within the same tank were pooled to form one biological replicate, resulting in three independent tank-derived biological replicates for each group.

In addition to spleen, intestine and kidney tissues were also collected for hematoxylin and eosin (H&E) staining, and spleen tissues were further processed for TUNEL staining. All tissues designated for histopathological and apoptosis analyses were fixed in 4% paraformaldehyde immediately after dissection. The remaining spleen samples were snap-frozen in liquid nitrogen and stored at −80 °C until further analysis.

### 2.2. H&E/TUNEL Staining Assays

Intestine, spleen, and kidney tissues (*n* = 3) were fixed in 4% paraformaldehyde solution for 24 h. Tissue sections were deparaffinized in xylene for 10 min and then rehydrated through a graded ethanol series. The rehydrated sections were stained with hematoxylin for 5 min to visualize nuclei. After rinsing with tap water, the sections were stained with eosin for 1 min to visualize the cytoplasm. For TUNEL staining, rehydrated spleen sections were first permeabilized with PBS containing 1% Triton X-100 for 10 min at room temperature and then digested with proteinase K at 37 °C. TUNEL staining was performed according to the manufacturer’s instructions using a TUNEL detection kit (KeyGEN, Nanjing, China). Finally, the stained sections were scanned and imaged using an Olympus VS200 digital slide scanning system (Olympus Corporation, Tokyo, Japan).

### 2.3. Measurement of Enzyme Activities and Immune Parameters

Spleen samples were collected from *A. fasciatus* at 0, 24, 48, and 72 h post-infection (hpi) for enzyme activity assays and immune parameter measurements. All time points included three independent biological replicates (one per tank). Malondialdehyde (MDA) levels were measured using the thiobarbituric acid (TBA) method according to the instructions of the commercial assay kits from Nanjing Jiancheng Bioengineering Institute, China. Total superoxide dismutase (T-SOD) activity was determined using the WST-1 method, and total antioxidant capacity (T-AOC) was measured using the ABTS method. The levels of complement component 3 (C3), lysozyme (LZM), and immunoglobulin M (IgM) were determined using enzyme-linked immunosorbent assay (ELISA) kits (NJJCBIO, Nanjing, China).

### 2.4. miRNA, lncRNA and circRNA Identification

Total RNA was extracted from spleen tissues using TRIzol reagent (Invitrogen, Carlsbad, CA, USA). After quality control, qualified RNA samples were selected for library construction. The small RNA sequencing (sRNA-seq) library construction and data processing procedures were performed as previously described [42]. Briefly, RNAs 18–30 nt in length were ligated with 3′ and 5′ adapters, followed by reverse transcription. PCR products of 140–160 bp were recovered by gel excision and used for library construction. Raw reads generated on the Illumina NovaSeq 6000 platform were subjected to quality control using fastp v0.12. 4. High-quality (HQ) reads were obtained after filtering and were then aligned against the GenBank sRNA database (release 209.0) and the Rfam database using blastall v2.2.25. The clean reads were also mapped to the *A. fasciatus* reference genome using HISAT2 v2. 2.1. Tags aligned to miRBase v21.0 were identified as known miRNAs, and novel miRNAs were predicted using MIREAP v0.2.

For lncRNA identification, high-quality reads were mapped to the *A. fasciatus* reference genome using HISAT2 v2.2.1. The protein-coding potential of transcripts longer than 200 nt was evaluated using three computational approaches: CNCI v2 [43], CPC v0.9-r2 [44], and FEELnc v0.2 [45]. Transcripts predicted by all three algorithms to lack protein-coding potential were identified as lncRNAs in *A. fasciatus*.

For circRNA identification, unmapped reads were collected from the alignment results. The terminal sequences of each unmapped read were then aligned to the *A. fasciatus* reference genome to identify unique anchor positions spanning splice sites. Reads showing head-to-tail alignment were analyzed using find_circ [35] to identify circRNAs in *A. fasciatus*.

### 2.5. Differential Expression Analysis

To quantify the expression abundance of mRNAs, miRNAs, and lncRNAs, transcripts per million (TPM) values for each transcript were calculated using RSEM. CircRNA abundance was normalized as reads per million mapped reads (RPM). Differentially expressed mRNAs (DEGs), miRNAs (DEmiRNAs), lncRNAs (DElncRNAs), and circRNAs (DEcircRNAs) between the control and infected groups were identified using the edgeR package v3.12.1 [46]. For DEGs and DElncRNAs, the screening threshold was set as false discovery rate *FDR* < 0.05 and |log_2_FC| > 1. For DEmiRNAs and DEcircRNAs, the screening threshold was set as *p* < 0.05 and |log_2_FC| > 1. Given the relatively low number of expressed miRNAs and circRNAs and the high sparsity of their expression matrices, strict FDR correction would be overly conservative and exclude bona fide immune-related regulatory molecules; thus, raw *p* values were used for preliminary screening. As a sensitivity check, we confirmed that the core immune-related pathways and key nodes of the ceRNA network remained consistently identified under an FDR < 0.1 threshold, supporting the robustness of the main conclusions.

### 2.6. Functional Enrichment Analysis

Functional enrichment analysis was performed using the Gene Ontology (GO) database [47]. Kyoto Encyclopedia of Genes and Genomes (KEGG) pathway enrichment analysis was conducted to identify significantly enriched biological pathways [48]. GO terms and KEGG pathways with an FDR < 0.05 were considered significantly enriched.

### 2.7. Identification of miRNA Target Genes

To identify potential target genes regulated by miRNAs, target prediction was performed using three algorithms: miRanda v3.3a, TargetScan v7.0, and RNAhybrid v2.1.2. Only targets predicted by at least two algorithms were retained as reliable miRNA target genes. Candidate genes showing negative expression correlations with DEmiRNAs were considered differentially expressed target genes (DETGs) of the corresponding DEmiRNAs. DEmiRNAs negatively correlated with DElncRNAs were regarded as target miRNAs of DElncRNAs, namely differentially expressed target miRNAs (DETmiRNAs).

### 2.8. Construction of the Competing Endogenous RNA (ceRNA) Network

Based on the cis- and trans-regulatory modes of lncRNAs, co-expression and co-localization analyses were performed to identify potential target genes of lncRNAs [45]. DEGs located within 10 kb upstream or downstream of DElncRNAs in the genome were identified as cis-regulated differentially expressed lncRNA target genes (cis-DELTGs). DEGs located more than 10 kb away from DElncRNAs and showing significant expression correlations with DElncRNAs were considered trans-regulated differentially expressed lncRNA target genes (trans-DELTGs). All cis- and trans-DELTGs were regarded as differentially expressed target genes of DElncRNAs.

The lncRNA-miRNA-mRNA ceRNA network was constructed according to the following criteria, with reference to previously reported procedures [45]:

1. DElncRNAs and DEGs with a significant positive expression correlation were retained as candidate ceRNA pairs (Pearson correlation coefficient > 0.8, *p* < 0.05);
2. Both the lncRNA and mRNA in each candidate pair were predicted to share at least one common miRNA response element (MRE) for the same DEmiRNA, with target prediction supported by at least two of the three algorithms (miRanda v3.3a, TargetScan v7.0, and RNAhybrid v2.1.2);
3. The expression level of the shared DEmiRNA was significantly negatively correlated with both the corresponding DElncRNA and DEmRNA (Pearson correlation coefficient < −0.7, *p* < 0.05).

To focus on core immune regulatory axes, we selected 10 immune-related DEGs for final network construction. These mRNAs were screened from significantly enriched immune KEGG pathways and met the criteria of |log_2_FC| > 2, FDR < 0.01. Triplets satisfying the above criteria were included in the final ceRNA network, which was constructed and visualized using Cytoscape v3.7.1. All regulatory relationships described in this network remain putative and bioinformatically predicted.

### 2.9. qRT-PCR Validation

To verify the cross-platform consistency of the RNA-seq expression trends, six DEGs, six DElncRNAs, and six DEmiRNAs were selected for qRT-PCR analysis. The RNA samples used for qRT-PCR were the same three biological replicates used for RNA-seq library preparation. The primers used for qRT-PCR are listed in Table S1. cDNA was synthesized using the HiFiScript gDNA Removal cDNA Synthesis Kit (Jiangsu Cowin Biotech Co., Ltd., Jiangsu, China), and qRT-PCR was performed on a Roche LightCycler^®^ 480 II system (Roche Diagnostics, Rotkreuz, Switzerland). *β-actin* was used as the internal reference gene for mRNAs and lncRNAs, whereas *U6* was used as the internal reference for miRNAs.

### 2.10. Statistical Analysis

Relative gene expression levels were calculated using the 2^−ΔΔCt^ method. Differences in gene expression were analyzed by one-way ANOVA followed by LSD post hoc test using SPSS v26.0. Differences in splenic enzyme activities and immune-related protein levels were evaluated by one-way analysis of variance (ANOVA) using SPSS v26.0. The degree of apoptosis detected by TUNEL staining was quantified using ImageJ 1.54f software. All values are presented as the mean ± standard deviation (SD). Statistical significance was indicated as follows: *p* < 0.05, *; *p* < 0.01, **; *p* < 0.001, ***; and *p* < 0.0001, ****.

## 3. Results

### 3.1. Histological Analysis of the Intestine, Spleen, and Kidney of A. fasciatus Following C. freundii Infection

**Figure 1:**
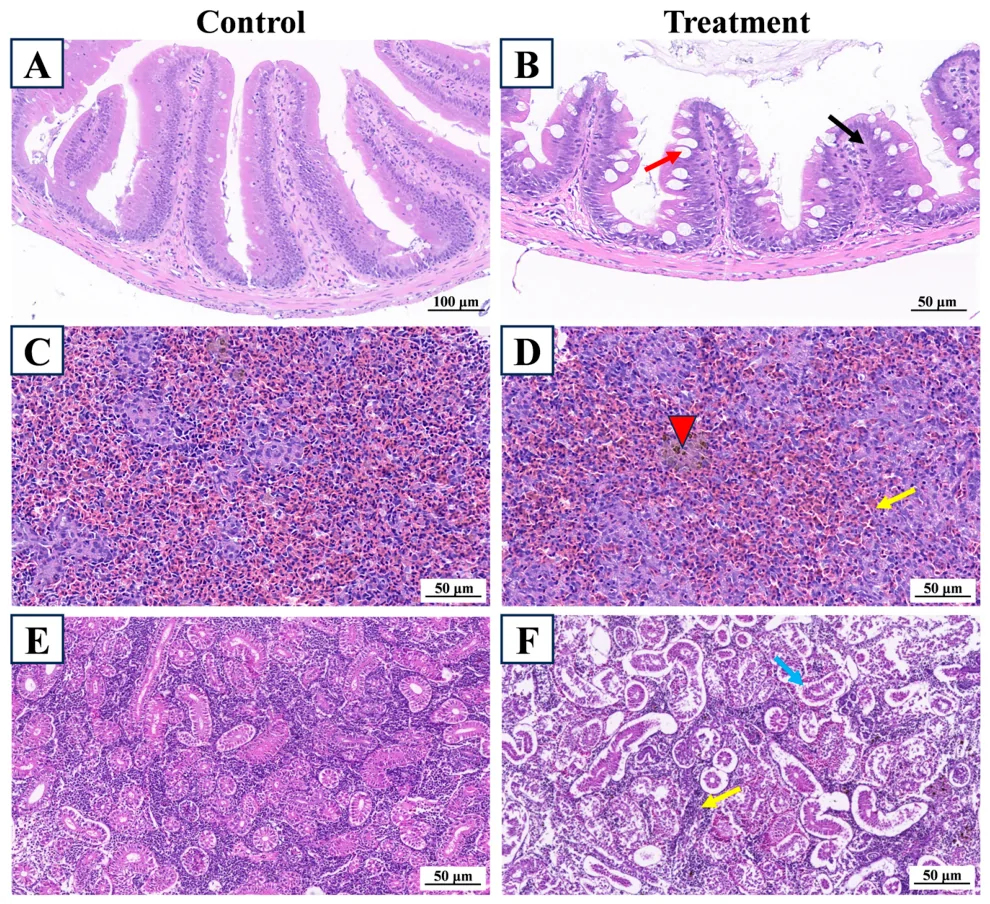
Histopathological changes in different tissues of *A. fasciatus* from the control and infected groups. (**A**,**B**), intestine; (**C**,**D**), spleen; (**E**,**F**), kidney. Red arrows indicate goblet cell vacuolization; black arrows indicate intestinal villus deformation; red triangles indicate hemosiderin deposition; yellow arrows indicate inflammatory cell infiltration; blue arrows indicate renal tubule deformation. (For interpretation of the references to colour in this figure legend, the reader is referred to the Web version of this article).

**Figure 2:**
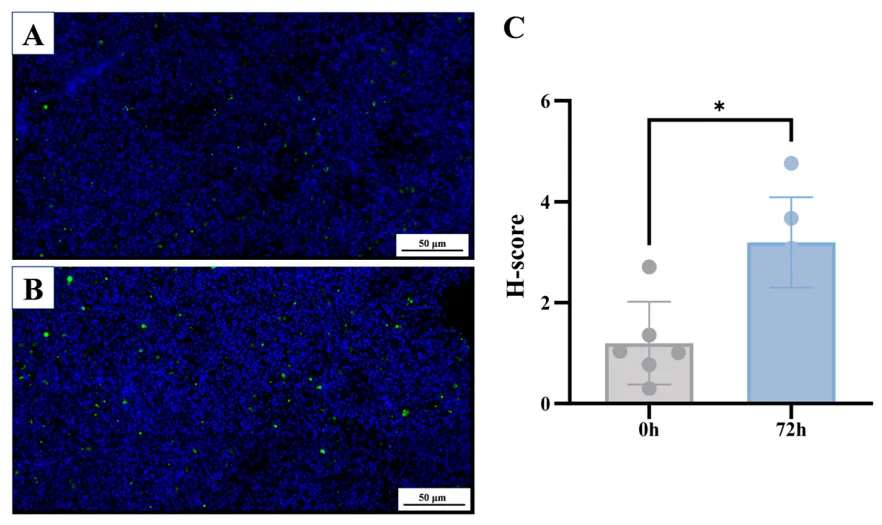
TUNEL staining was performed to assess splenic apoptotic cells. (**A**) 0 h group; (**B**) 72 h post-infection group. (**C**) H-score quantification of TUNEL staining. Values are mean ± SD. *p*< 0.05, *.

In the control group, the intestine exhibited a clear and intact histological structure, with well-organized intestinal villi, numerous goblet cells distributed along the mucosal surface, and abundant intestinal villi (Figure 1A). In the infected group, the intestinal villi were deformed, the local epithelial arrangement was slightly disordered, and prominent goblet cells with vacuolization were observed (Figure 1B). The spleen of the control group showed an intact structure with abundant lymphocytes (Figure 1C). In contrast, punctate necrosis, inflammatory cell infiltration, and hemosiderin deposition were observed in the spleen of the infected group (Figure 1D). The kidney in the control group displayed a normal overall structure, and the renal tubules were generally intact (Figure 1E). After infection, deformation of renal tubules and inflammatory cell infiltration were observed in the kidney (Figure 1F). TUNEL staining was performed to verify whether *C. freundii* infection induced splenic cell death (Figure 2A,B). The proportion of apoptotic cells in the spleen was significantly increased after *C. freundii* infection (Figure 2C).

### 3.2. Effects of C. freundii Infection on Enzyme Activities and Immune-Related Factors in A. fasciatus

**Figure 3:**
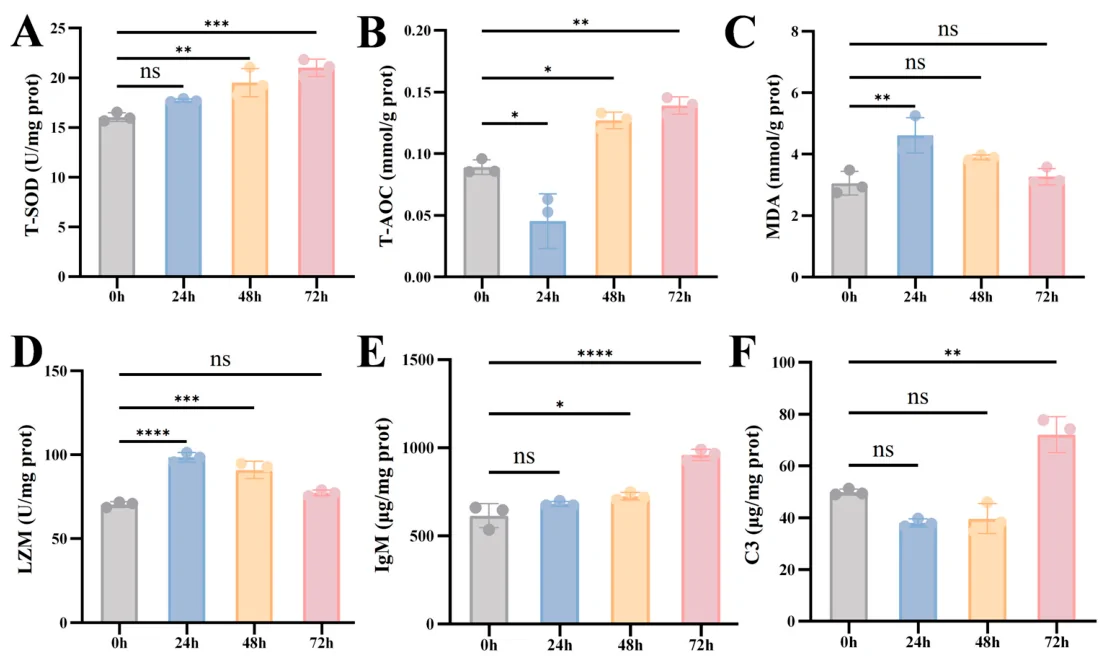
Effects of *C. freundii* infection on splenic enzyme activities and immune-related factors in *A. fasciatus*. (**A**), total superoxide dismutase (T-SOD); (**B**), total antioxidant capacity (T-AOC); (**C**), malondialdehyde (MDA); (**D**), lysozyme (LZM); (**E**), immunoglobulin M (IgM); (**F**), complement component 3 (C3). Data are presented as the mean ± SD. Statistical significance is indicated as follows: *p* < 0.05, *; *p* < 0.01, **; *p* < 0.001, ***; and *p* < 0.0001, ****.

T-SOD activity in *A. fasciatus* showed an increasing trend after *C. freundii* infection and was significantly higher at 48 and 72 h than at 0 h (Figure 3A). T-AOC decreased significantly at 24 h, followed by a marked increase at 48 and 72 h (Figure 3B). In contrast, MDA content increased at 24 h and then declined thereafter (Figure 3C). LZM activity increased significantly and reached its peak at 24 h but began to decrease after 48 h (Figure 3D). IgM content gradually increased with infection time and was significantly elevated at 48 and 72 h (Figure 3E). C3 content decreased slightly at 24 and 48 h, followed by a significant increase at 72 h (Figure 3F).

### 3.3. Identification and Characterization of lncRNAs in A. fasciatus Following C. freundii Infection

**Figure 4:**
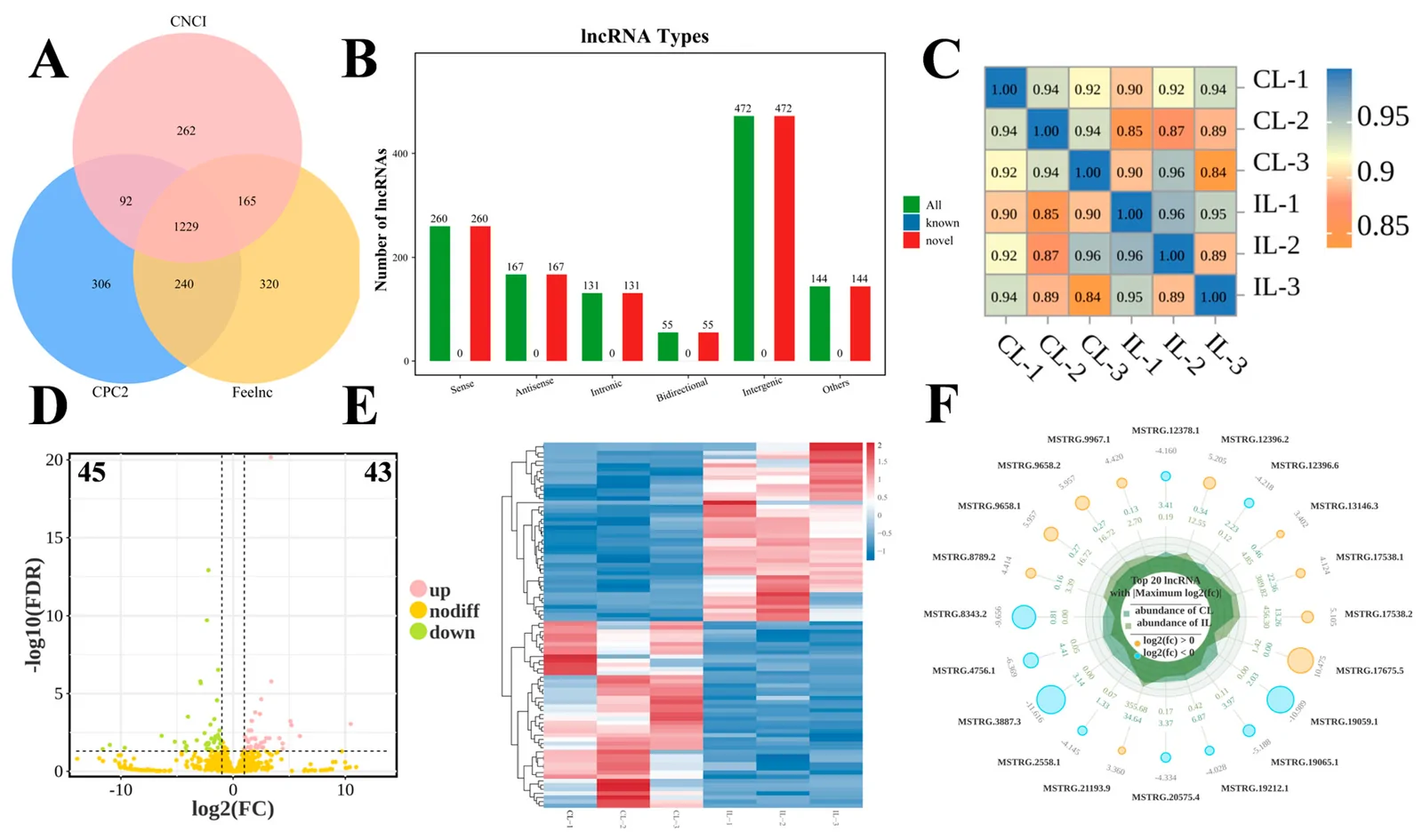
Identification and characterization of lncRNAs in *A. fasciatus* following *C. freundii* infection. (**A**) Venn diagram showing the coding potential assessment of lncRNAs using CPC2, CNCI, and FEELnc. (**B**) Classification of lncRNAs. (**C**) Pearson correlation heatmap of samples. (**D**) Volcano plot of differentially expressed lncRNAs. (**E**) Expression patterns of DElncRNAs. (**F**) Radar plot of the top 20 upregulated and downregulated DElncRNAs.

Sequencing data from six libraries, including three control libraries and three infected libraries, were analyzed to identify lncRNAs. Because lncRNAs lack protein-coding potential, three coding potential analysis tools, namely CPC2, CNCI, and FEELnc, were used to evaluate transcript coding capacity. Based on the overlapping results, a total of 1229 lncRNAs were identified after *C. freundii* infection (Figure 4A). According to their genomic locations, these lncRNAs were classified into six categories: 260 sense lncRNAs, 167 antisense lncRNAs, 131 intronic lncRNAs, 55 bidirectional lncRNAs, 472 intergenic lncRNAs, and 144 other lncRNAs (Figure 4B). The Pearson correlation heatmap showed high consistency among biological replicates within each group, whereas samples from the control and infected groups were clearly separated (Figure 4C). A total of 88 DElncRNAs, including 43 upregulated and 45 downregulated lncRNAs, were identified after infection (Figure 4D). The clustering heatmap further illustrated the expression patterns of these DElncRNAs (Figure 4E). The top 20 upregulated and downregulated DElncRNAs after infection are shown in a radar plot (Figure 4F).

### 3.4. Identification and Characterization of circRNAs in A. fasciatus Following C. freundii Infection

**Figure 5:**
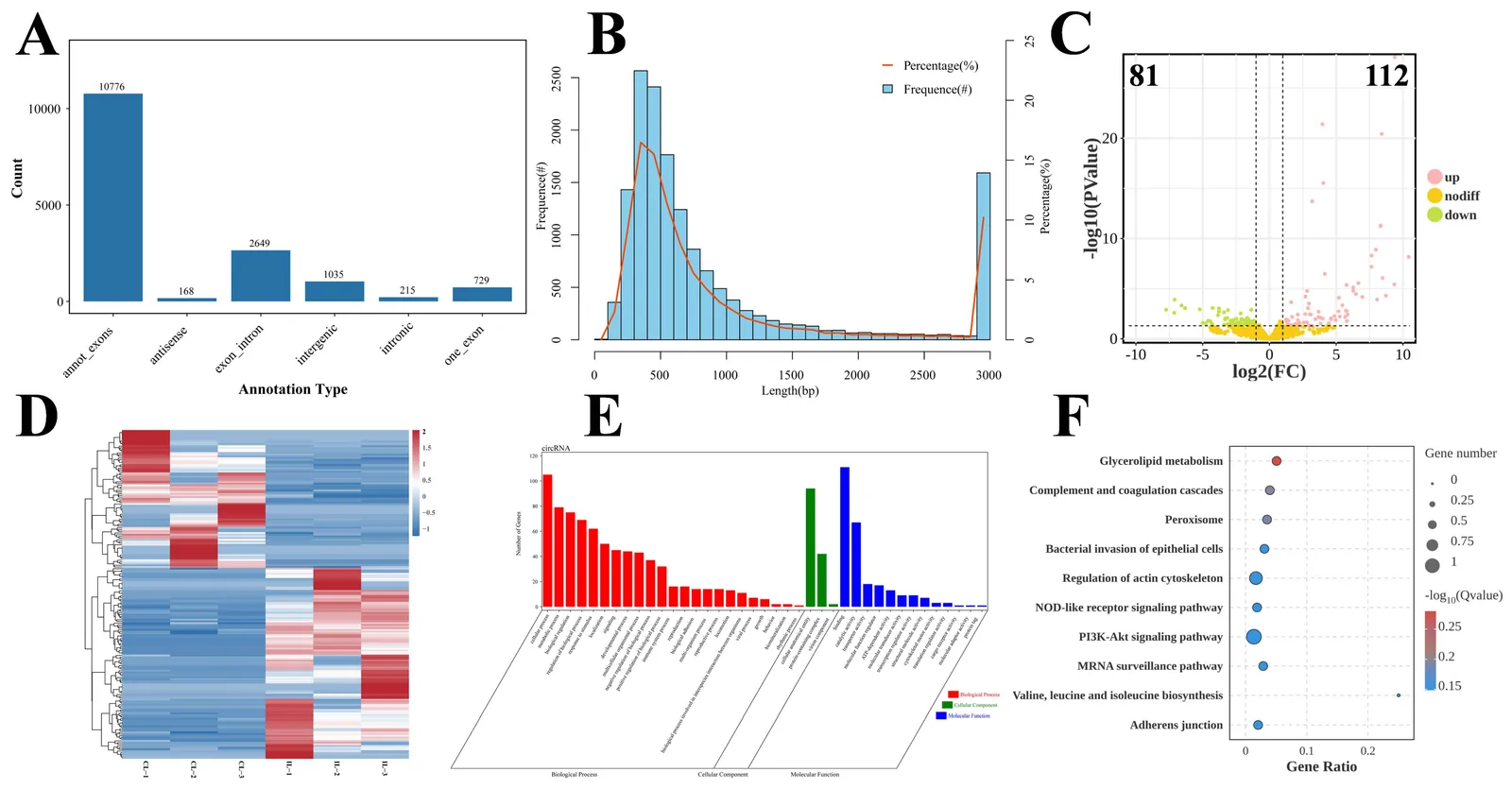
Identification and characterization of circRNAs in *A. fasciatus* following *C. freundii* infection. (**A**) Classification of circRNAs. (**B**) Length distribution of circRNAs. (**C**) Volcano plot of differentially expressed circRNAs. (**D**) Expression patterns of DEcircRNAs. (**E**) GO enrichment analysis of DEcircRNA host genes. (**F**) KEGG pathway enrichment analysis of DEcircRNA host genes.

To identify circRNAs in *A. fasciatus* following *C. freundii* infection, unmapped reads were collected and circRNAs were screened using find_circ. A total of 15,572 circRNAs were identified after infection. According to their structural origin, these circRNAs were classified into six categories: annotated exonic, antisense, exon–intron, intergenic, intronic, and one-exon circRNAs (Figure 5A). The length distribution histogram showed that most circRNAs were 300–500 bp in length (Figure 5B). A total of 193 DEcircRNAs, including 112 upregulated and 81 downregulated circRNAs, were identified after infection (Figure 5C). The expression patterns of DEcircRNAs are shown in Figure 5D. GO enrichment analysis showed that the host genes of DEcircRNAs were mainly enriched in cellular process, metabolic process, biological regulation, regulation of biological process, and response to stimulus (Figure 5E). KEGG pathway enrichment analysis revealed that DEcircRNAs were significantly enriched in immune-related pathways, including complement and coagulation cascades, NOD-like receptor signaling pathway, bacterial invasion of epithelial cells, and PI3K-Akt signaling pathway (Figure 5F).

### 3.5. Identification and Characterization of miRNAs in A. fasciatus Following C. freundii Infection

**Figure 6:**
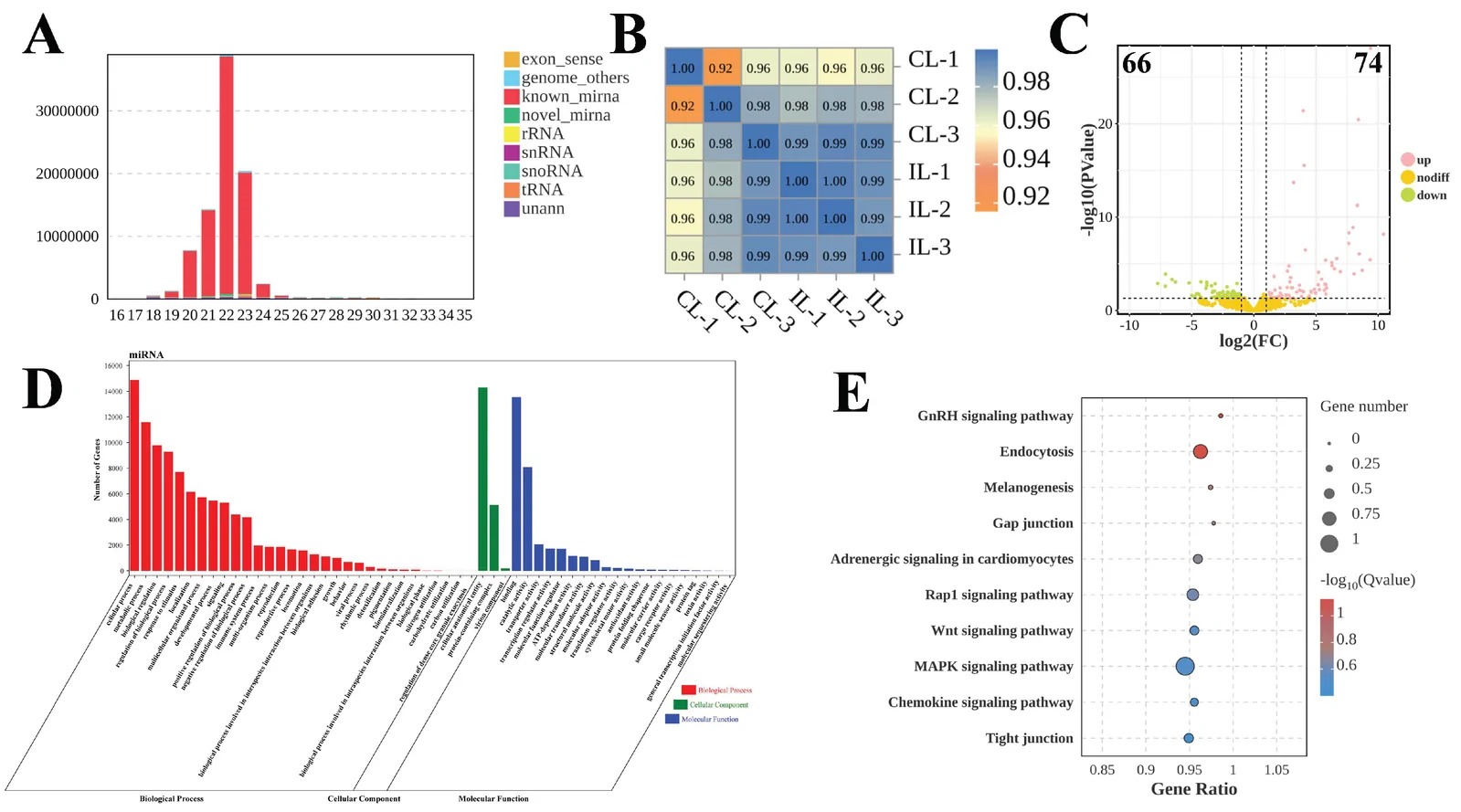
Identification and characterization of miRNAs in *A. fasciatus* following *C. freundii* infection. (**A**) Length distribution of miRNAs. (**B**) Pearson correlation heatmap of samples. (**C**) Volcano plot of differentially expressed miRNAs. (**D**) GO enrichment analysis of DEmiRNA target genes. (**E**) KEGG pathway enrichment analysis of DEmiRNA target genes.

To identify miRNAs, high-quality reads were first aligned against the sRNA and Rfam databases and then mapped to the *A. fasciatus* reference genome. Tags aligned to miRBase were identified as known miRNAs, whereas novel miRNAs were predicted using MIREAP. The length distribution histogram showed that most miRNAs ranged from 20 to 24 nt, with 22 nt miRNAs accounting for the largest proportion (Figure 6A). The Pearson correlation heatmap showed high consistency among biological replicates within each group, whereas samples from the control and infected groups were clearly separated (Figure 6B). A total of 140 DEmiRNAs, including 74 upregulated and 66 downregulated miRNAs, were identified in the infected group compared with the control group (Figure 6C). GO enrichment analysis showed that the target genes of DEmiRNAs were mainly enriched in cellular process, metabolic process, biological regulation, response to stimulus, and regulation of biological process (Figure 6D). KEGG pathway enrichment analysis revealed that the target genes of DEmiRNAs were significantly enriched in immune-related pathways, including endocytosis, MAPK signaling pathway, and chemokine signaling pathway (Figure 6E).

### 3.6. Identification and Characterization of mRNAs in A. fasciatus Following C. freundii Infection

**Figure 7:**
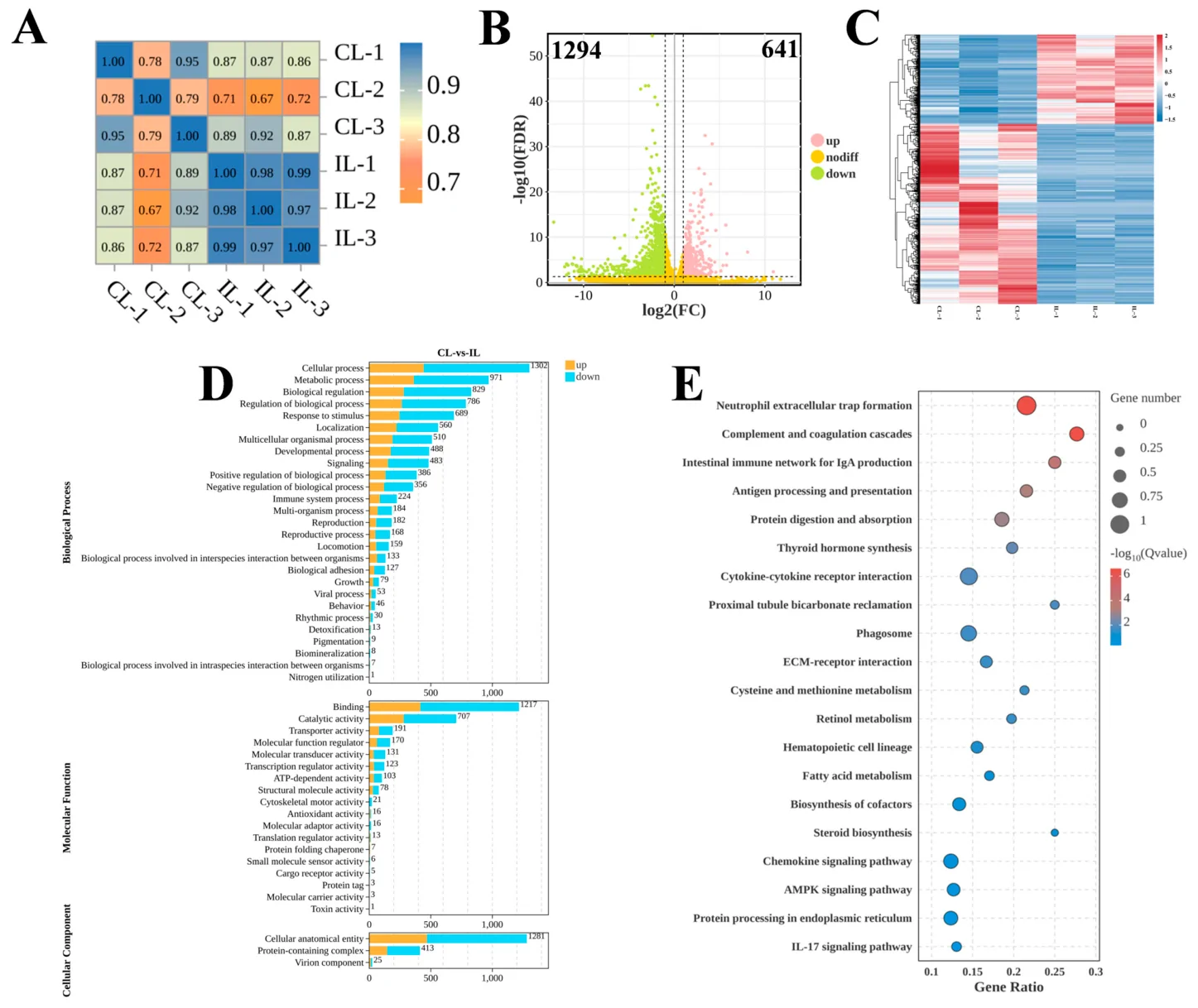
Identification and analysis of mRNAs in *A. fasciatus* following *C. freundii* infection. (**A**) Pearson correlation heatmap of samples. (**B**) Volcano plot of differentially expressed genes. (**C**) Clustering heatmap of DEG expression. (**D**) GO enrichment classification of DEGs. (**E**) KEGG pathway enrichment analysis of DEGs.

Pearson correlation analysis and a DEG volcano plot were used to characterize transcriptomic differences between the control and infected groups. The Pearson correlation heatmap showed high similarity among biological replicates, indicating good reproducibility of the sequencing data (Figure 7A). A total of 1935 DEGs, including 1294 downregulated and 641 upregulated genes, were identified after *C. freundii* infection (Figure 7B). The expression patterns of DEGs are shown in Figure 7C. GO enrichment analysis showed that DEGs were mainly enriched in cellular process, metabolic process, biological regulation, response to stimulus, and catalytic activity (Figure 7D). KEGG pathway enrichment analysis revealed that DEGs were significantly enriched in immune-related pathways, including neutrophil extracellular trap formation, complement and coagulation cascades, antigen processing and presentation, cytokine–cytokine receptor interaction, phagosome, chemokine signaling pathway, and IL-17 signaling pathway (Figure 7E).

### 3.7. Expression Patterns of Immune-Related Differentially Expressed mRNAs

**Figure 8:**
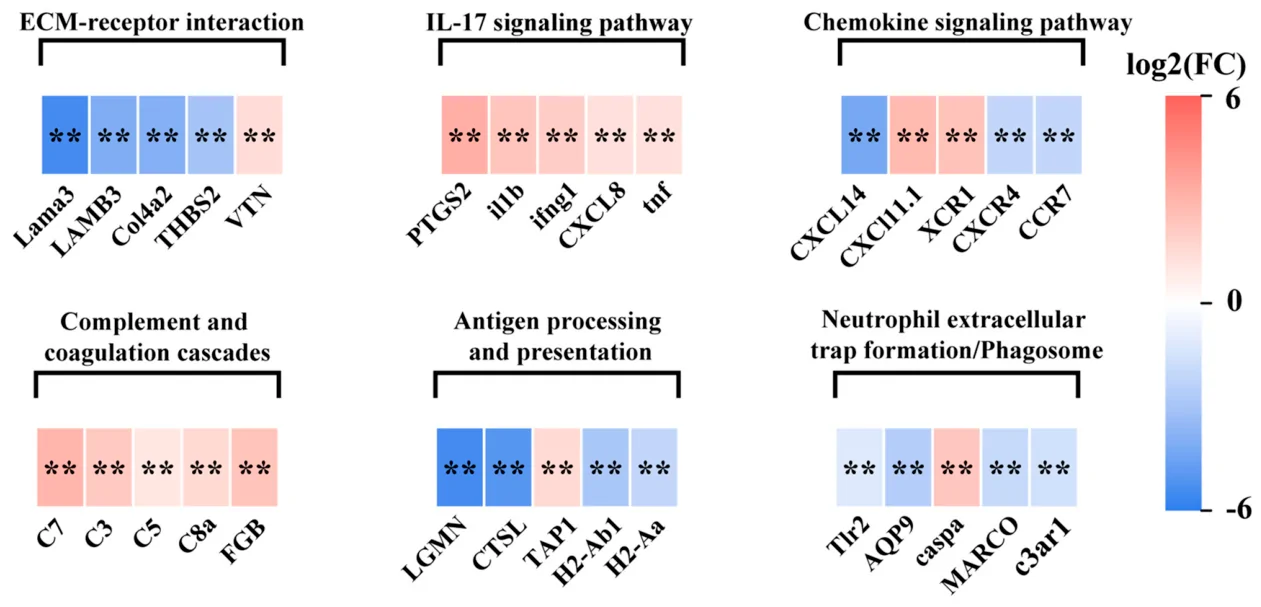
Heatmap of expression levels for 30 differentially expressed mRNAs. *p* < 0.01, **.

A total of 30 differentially expressed genes from seven KEGG pathways were selected for expression analysis (Figure 8 and Table S2). Among genes associated with the ECM-receptor interaction pathway, *Lama3*, *LAMB3*, *Col4a2*, and *THBS2* were significantly downregulated (*p* < 0.01), whereas *VTN* was significantly upregulated (*p* < 0.01). In the IL-17 signaling pathway, *PTGS2*, *il1b*, *ifng1*, *CXCL8*, and *tnf* were significantly upregulated (*p* < 0.01). In the chemokine signaling pathway, *CXCL11.1* and *XCR1* were significantly upregulated (*p* < 0.01), whereas *CXCL14*, *CXCR4*, and *CCR7* were significantly downregulated (*p* < 0.01). Among genes involved in the complement and coagulation cascades, *C7*, *C3*, *C5*, *C8a*, and *FGB* were significantly upregulated (*p* < 0.01). In the antigen processing and presentation pathway, *TAP1* was significantly upregulated (*p* < 0.01), whereas *LGMN*, *CTSL*, *H2-Ab1*, and *H2-Aa* were significantly downregulated (*p* < 0.01). Among genes associated with neutrophil extracellular trap formation and phagosome pathways, *caspa* was significantly upregulated (*p* < 0.01), whereas *Tlr2*, *AQP9*, *c3ar1*, and *MARCO* were significantly downregulated (*p* < 0.01).

### 3.8. LncRNA-Mediated ceRNA Network

**Figure 9:**
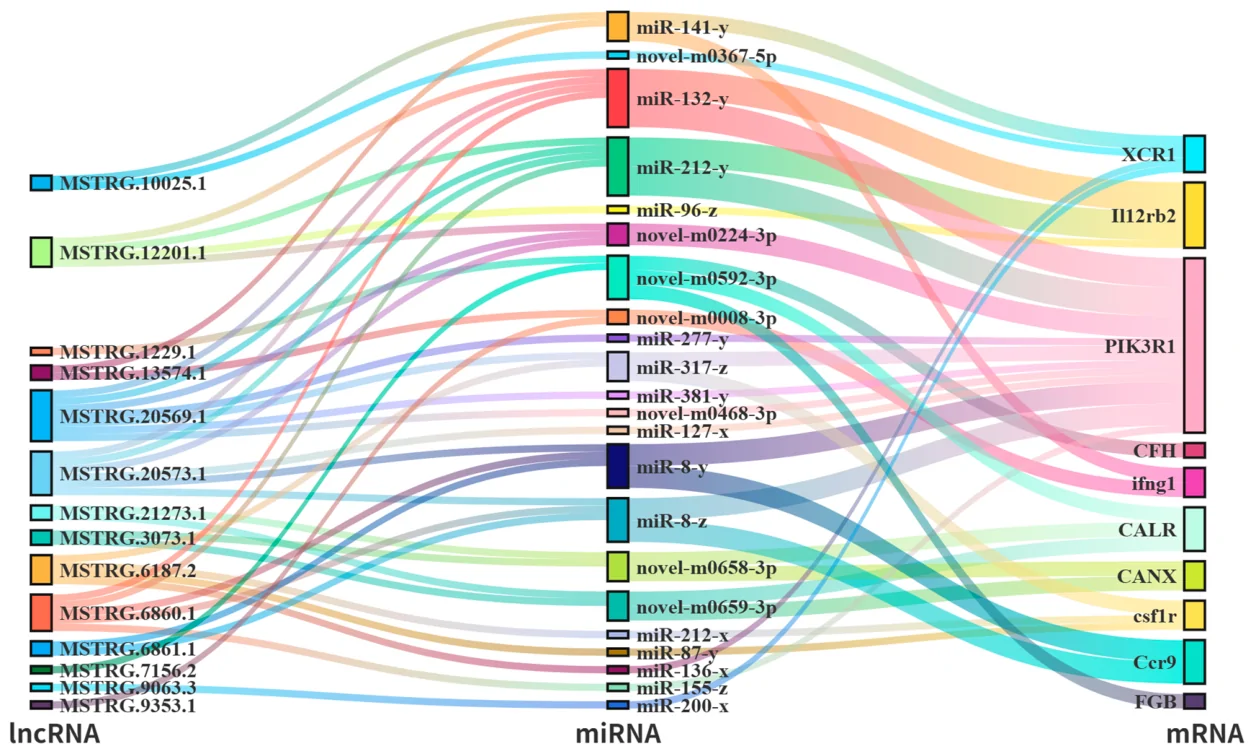
LncRNA-miRNA-mRNA ceRNA regulatory network. Nodes on the **left** represent differentially expressed lncRNAs, nodes in the **middle** represent miRNAs, and nodes on the **right** represent target mRNAs.

LncRNAs can act as ceRNAs by competitively binding to miRNAs through shared miRNA response elements, thereby regulating the expression of miRNA-targeted mRNAs. Based on the immune-related KEGG pathways enriched by the differentially expressed mRNAs, 10 mRNAs were selected to construct a lncRNA-miRNA-mRNA co-expression network. The resulting network comprised 10 mRNAs, 14 lncRNAs, and 22 miRNAs, forming 44 putative regulatory interactions (Figure 9). Within this network, multiple lncRNAs were predicted to interact with specific miRNAs and may regulate the expression of downstream immune-related mRNAs in a miRNA-mediated manner. Specifically, *MSTRG.12201.1*, *MSTRG.20569.1*, *MSTRG.20573.1*, and *MSTRG.6860.1* were predicted to share binding with *miR-212-y*, which in turn targeted *Il12rb2* and *PIK3R1*. In addition, *MSTRG.1229.1*, *MSTRG.7156.2* were predicted to bind *novel-m0592-3p* and potentially regulate the downstream targets *CFH*, *CALR*, and *FGB*. Moreover, *MSTRG.20573.1*, *MSTRG.6860.1*, and *MSTRG.6861.1* may regulate *Ccr9* and *PIK3R1* by interacting with *miR-8-y*.

### 3.9. qRT-PCR Validation

**Figure 10:**
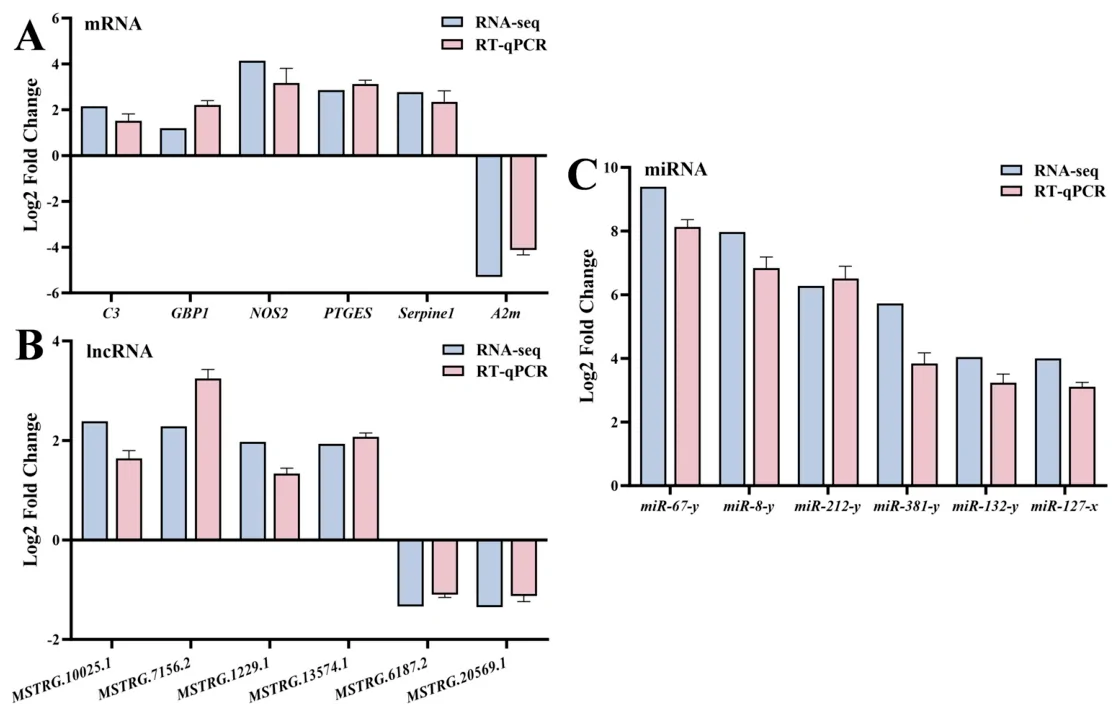
Validation of RNA-seq data by qRT-PCR. Relative expression patterns of selected DEGs, DElncRNAs, and DEmiRNAs were determined by RNA-seq (blue bars) and qRT-PCR (pink bars). (**A**) mRNAs; (**B**) lncRNAs; (**C**) miRNAs. qRT-PCR data are presented as the mean ± SD of three biological replicates. Relative expression levels in qRT-PCR were calculated using the 2^−ΔΔCt^ method. (For interpretation of the references to colour in this figure legend, the reader is referred to the Web version of this article).

To validate the reliability of the RNA-seq data, six DEGs, six DElncRNAs, and six DEmiRNAs were randomly selected for qRT-PCR analysis (Figure 10). The selected transcripts included *Serpine1*, *PTGES*, *GBP1*, *C3*, *NOS2*, *A2m*, *MSTRG.10025.1*, *MSTRG.20569.1*, *MSTRG.1229.1*, *MSTRG.13574.1*, *MSTRG.6187.2*, *MSTRG.7156.2*, *miR-132-y*, *miR-67-y*, *miR-8-y*, *miR-212-y*, *miR-127-x*, and *miR-381-y*. The expression trends of all 18 selected transcripts were consistent with the RNA-seq results, supporting the cross-platform reliability of the transcriptome profiling at the technical level.

## 4. Discussion

**Figure 11:**
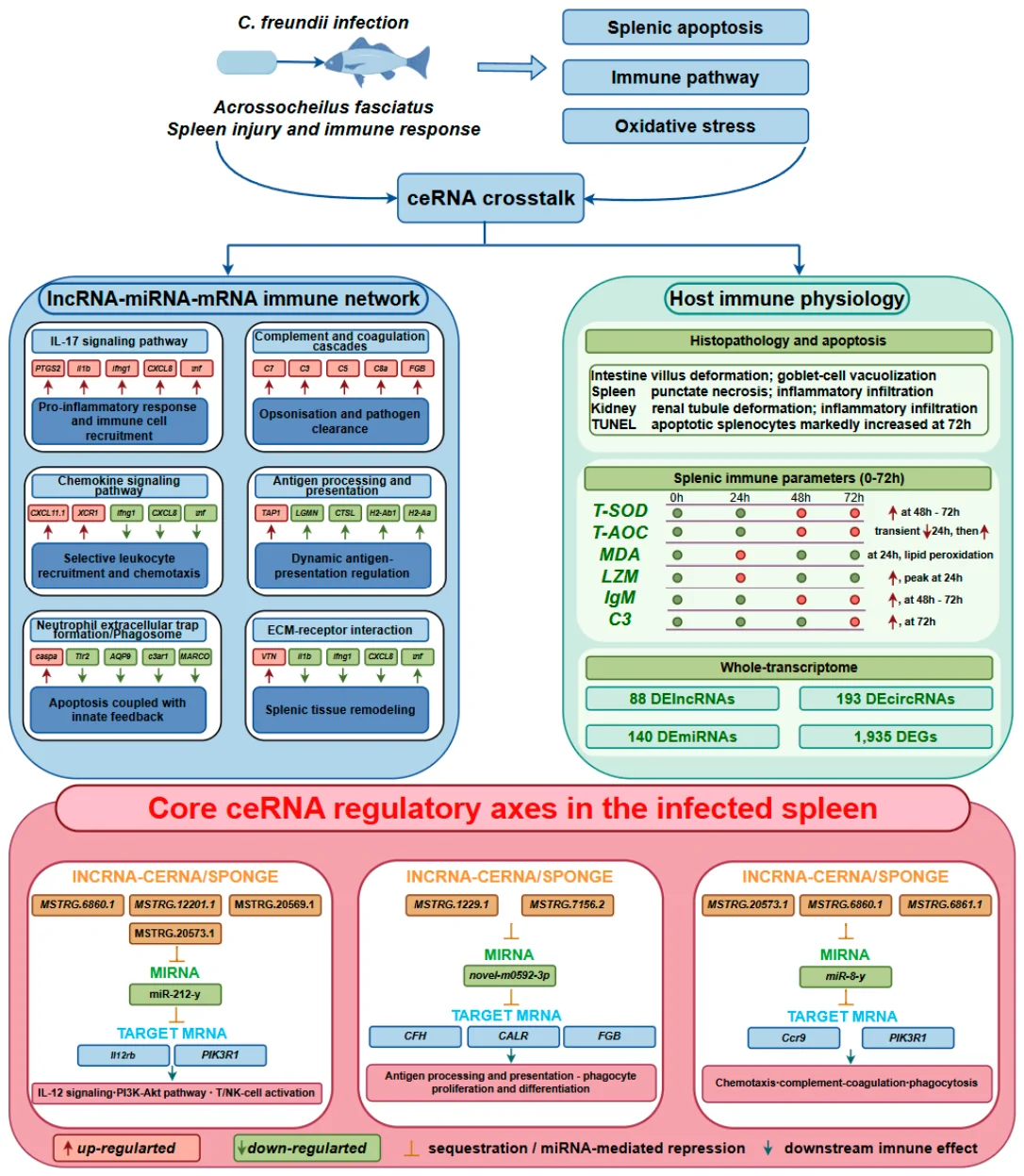
Schematic model of lncRNA-mediated ceRNA regulatory network in the spleen of *A. fasciatus* upon *C. freundii* infection.

In this study, histopathological analysis, apoptosis detection, immune physiological assays, and whole-transcriptome sequencing were integrated to systematically characterize the immune regulatory features of the spleen in *A. fasciatus* following *C. freundii* infection (Figure 11). The constructed lncRNA-miRNA-mRNA regulatory network identified key immune-related genes associated with the response of *A. fasciatus* to *C. freundii* infection and may facilitate the discovery of potential biomarkers for improving disease resistance in fish. In addition, putative ceRNAs were identified, which may competitively bind shared miRNAs and thereby influence the expression of corresponding target genes.

### 4.1. Pathogenic Effects of C. freundii Infection on A. fasciatus

Bacterial diseases caused by pathogens related to the order Enterobacterales have become major constraints on the healthy development of aquaculture [49,50,51]. In the present study, *A. fasciatus* infected with *C. freundii* showed typical clinical symptoms, including hemorrhage and redness in the abdomen and fins, as well as congestion and enlargement of the liver and spleen (Figure S2). Histopathological examination further revealed marked tissue damage in the intestine, spleen, and kidney after infection (Figure 1). The intestine is an essential organ for digestion, nutrient absorption, and maintenance of mucosal immune homeostasis in fish, and its structural integrity directly affects nutrient utilization, barrier function, and growth performance [52,53,54]. When pathogen infection induces intestinal villus damage, epithelial disorganization, or barrier dysfunction, feeding behavior, digestive and absorptive capacity, and metabolic homeostasis may be impaired, thereby contributing to anorexia, energy metabolism disturbance, and growth inhibition [55]. The kidney is also an important organ involved in excretion, osmoregulation, and immune defense in fish [56,57,58]. In this study, renal tubule deformation and inflammatory cell infiltration were observed in the kidney of infected *A. fasciatus*, suggesting that *C. freundii* infection may damage renal tissue structure and further activate local inflammatory responses [59,60]. The spleen is a major immune and hematopoietic organ in fish and participates in antigen clearance, immune cell proliferation, and regulation of inflammatory responses [61,62]. After infection, punctate necrosis, inflammatory cell infiltration, and hemosiderin deposition were observed in the spleen of *A. fasciatus*. The increased proportion of TUNEL-positive cells in the spleen further confirmed tissue injury induced by *C. freundii* infection.

### 4.2. Immune Physiological Responses of A. fasciatus to C. freundii Infection

SOD can scavenge reactive oxygen species (ROS) generated during pathogen infection [63]. Moderate ROS production contributes to pathogen clearance by the host, whereas excessive ROS can induce lipid peroxidation, protein oxidation, and nucleic acid damage. Therefore, timely activation of the antioxidant system is essential for maintaining normal immune responses. T-AOC reflects the overall antioxidant capacity of an organism, while MDA, as one of the major products of lipid peroxidation, can partially indicate the extent of free radical-mediated membrane lipid oxidative damage [64]. In the present study, T-SOD activity showed an overall increasing trend after infection. Although T-AOC transiently decreased at 24 h, it increased markedly at 48 and 72 h. These results suggest that pathogen stimulation during the early stage of infection may rapidly induce ROS accumulation and consume part of the antioxidant reserves. Subsequently, the host may enhance SOD activity and overall antioxidant capacity to eliminate excessive ROS and alleviate oxidative damage. The increase in MDA content at 24 h further indicates that *C. freundii* infection induced oxidative stress and lipid peroxidation during the early stage of infection.

The innate immune system is an important defense line against pathogen invasion in fish [61]. In this study, LZM activity increased significantly at 24 h after infection, indicating that *A. fasciatus* rapidly initiated lysozyme-mediated antibacterial responses during the early stage of infection. The subsequent decline in LZM activity may be associated with immune consumption or pathogen immune evasion [65,66]. IgM and C3 are important components of humoral immunity and the complement system in fish, respectively [67,68]. The overall increase in IgM and the significant elevation of C3 at 72 h suggest that *A. fasciatus* further enhanced antibody- and complement-mediated humoral immune responses during the later stage of infection.

### 4.3. Disruption of Immune Homeostasis in A. fasciatus Following C. freundii Infection

Transcriptome analysis of the spleen of *A. fasciatus* after *C. freundii* infection identified a total of 1935 DEGs (Figure 7B). KEGG enrichment analysis showed that these DEGs were mainly involved in immune-related pathways, including neutrophil extracellular trap formation, complement and coagulation cascades, antigen processing and presentation, cytokine–cytokine receptor interaction, phagosome, chemokine signaling pathway, and IL-17 signaling pathway (Figure 7E). Previous studies have also reported significant enrichment of DEGs in these immune-associated pathways during bacterial infection in fish [69,70,71]. To further characterize the immune response of *A. fasciatus* to *C. freundii* infection, 30 differentially expressed mRNAs from seven representative pathways were selected for expression analysis (Figure 8).

Cytokines play important roles in pathogen recognition, inflammatory amplification, and immune cell communication in fish [72]. In this study, *PTGS2*, *il1b*, *ifng1*, *CXCL8*, and *tnf* were significantly upregulated, indicating that *C. freundii* infection induced a strong pro-inflammatory response and promoted immune cell recruitment. The complement system is an important humoral immune defense mechanism in teleost fish and participates in antibacterial immunity through opsonization, chemotaxis, and cell lysis [73]. The significant upregulation of *C3*, *C5*, *C7*, *C8a*, and *FGB* suggests that complement activation and coagulation responses jointly contributed to pathogen clearance after infection. Moreover, the coordinated upregulation of complement-related genes was consistent with the increased C3 content, further indicating that the complement system may be a key pathway involved in the defense of *A. fasciatus* against *C. freundii* infection.

Chemokine signaling-related genes showed selective expression changes after infection. *CXCL11.1* and *XCR1* were significantly upregulated, suggesting that *A. fasciatus* may enhance the chemotactic recruitment of specific immune cells and intercellular communication after infection. In contrast, *CXCL14*, *CXCR4*, and *CCR7* were significantly downregulated, indicating that some chemokine axes may be suppressed or dynamically regulated. Chemokines play important roles in immune cell migration, inflammatory localization, and anti-infective responses in teleost fish [74]. Therefore, the differential expression of these genes may reflect dynamic regulation of immune cell recruitment and inflammatory intensity in the host.

Genes associated with neutrophil extracellular trap formation and phagosome pathways also exhibited divergent expression patterns. *caspa* was significantly upregulated, which was consistent with the increased splenic cell apoptosis observed by TUNEL staining, suggesting that cell death may participate in immune regulation and tissue injury after infection. Conversely, *Tlr2*, *AQP9*, *c3ar1*, and *MARCO* were downregulated, suggesting that some processes related to pathogen recognition, complement receptor signaling, and phagocytosis may be inhibited or feedback-regulated. NET formation and phagocytic responses are important innate immune mechanisms that restrict bacterial dissemination [75,76]. These changes may therefore reflect coordinated regulation between antibacterial defense and inflammation-associated tissue damage in *A. fasciatus* following *C. freundii* infection.

### 4.4. Roles of Non-Coding RNAs in the Response of A. fasciatus to C. freundii Infection

Non-coding RNAs are widely involved in various biological processes, including precursor RNA processing, genome remodeling, cell development, and differentiation [20,21,22,23]. In this study, lncRNAs, circRNAs, and miRNAs in *A. fasciatus* following *C. freundii* infection were systematically identified, and a lncRNA-miRNA-mRNA regulatory network was constructed. After infection, a total of 88 DElncRNAs, 193 DEcircRNAs, and 140 DEmiRNAs were identified. Previous studies have demonstrated that lncRNAs, circRNAs, and miRNAs can participate in fish pathogen infection, inflammatory responses, and apoptosis by regulating target gene expression [24,25,26,27,33,34,36,37].

CircRNAs, as another important class of non-coding regulators with ceRNA potential, also showed extensive expression changes upon *C. freundii* infection. KEGG enrichment analysis of their host genes revealed significant enrichment in immune-related pathways including complement and coagulation cascades, NOD-like receptor signaling, bacterial invasion of epithelial cells, and PI3K-Akt signaling, indicating that circRNAs are also likely to participate in splenic immune regulation during bacterial infection. CircRNA-associated ceRNA networks and their specific immune regulatory roles will be systematically investigated in our follow-up work.

LncRNAs participate in the regulation of many biological processes through multiple mechanisms [77]. They can function as ceRNAs to regulate mRNAs and play crucial roles in host immune responses [78]. In this study, a lncRNA-miRNA-mRNA ceRNA regulatory network was constructed based on immune-related KEGG pathways enriched by differentially expressed mRNAs. Similar lncRNA-miRNA-mRNA networks have been shown to participate in pathogen infection responses in aquatic animals such as Japanese flounder, yellow catfish, pearl oyster, and leopard coral grouper [39,40,45,79], suggesting that ceRNA regulation may be an important regulatory mechanism in anti-infective immunity in aquatic animals.

In the present network, *MSTRG.12201.1*, *MSTRG.20569.1*, *MSTRG.20573.1*, and *MSTRG.6860.1* may jointly bind *miR-212-y* and further regulate *Il12rb2* and *PIK3R1*. *Il12rb2* is an important component of IL-12 receptor signaling and is closely associated with T cell- and NK cell-related immune responses. *PIK3R1* encodes a regulatory subunit of PI3K and participates in cell survival, metabolism, inflammatory signaling, and immune cell activation [80,81]. Therefore, these regulatory axes may connect cytokine signaling with PI3K-related immune transduction and represent candidate pathways involved in regulating inflammatory responses and immune cell activation after infection in *A. fasciatus*. Similarly, *MSTRG.20573.1*, *MSTRG.6860.1*, and *MSTRG.6861.1* may regulate *Ccr9* and *PIK3R1* through *miR-8-y*. *Ccr9* belongs to the chemokine receptor family and is mainly involved in immune cell migration and mucosal immune localization. The *CCL25/CCR9* axis has been shown to regulate the recruitment of T cells to specific sites [82]. Therefore, this ceRNA regulatory axis may participate in immune cell recruitment, inflammatory regulation, and pathogen clearance in *A. fasciatus* following *C. freundii* infection. *miR-212-y* and *miR-8-y* may be key miRNAs involved in the response of *A. fasciatus* to *C. freundii* infection.

### 4.5. Study Limitations and Future Perspectives

This study has several limitations that should be acknowledged. First, the ceRNA regulatory axes and key non-coding RNAs identified here are based on bioinformatic prediction and expression correlation analysis; their direct regulatory relationships and functional roles in antibacterial immunity have not been experimentally validated via gene knockdown/overexpression or dual-luciferase reporter assays. Second, the candidate molecules are associated with infection response, but their causal relationship with disease resistance or survival outcome has not been established in genetic populations or field breeding conditions. Third, only one post-infection time point (72 hpi) was selected for whole-transcriptome sequencing, which cannot fully capture the dynamic regulatory features across the entire infection process.

Future studies will focus on: (1) functional validation of the miR-212-y/*Il12rb2* and miR-8-y/*Ccr9* regulatory axes at the cellular and individual levels; (2) evaluation of the polymorphism and breeding value of candidate markers in natural or breeding populations; and (3) time-series transcriptomic analysis to reveal the dynamic ceRNA regulatory landscape during *C. freundii* infection.

## 5. Conclusions

In summary, this study provides the first integrated analysis of lncRNAs, circRNAs, miRNAs, and mRNAs in *A. fasciatus* following *C. freundii* infection. A total of 88 lncRNAs, 193 circRNAs, 140 miRNAs, and 1935 mRNAs were identified as differentially expressed after infection. The constructed lncRNA-miRNA-mRNA ceRNA network identified several potential regulatory axes, particularly those mediated by *miR-212-y* and *miR-8-y*. These findings provide a basis for understanding the molecular mechanisms underlying the antibacterial immune response of *A. fasciatus* and offer candidate targets for screening molecular markers for disease resistance research.

## Data Availability

The raw sequencing data generated in this study have been deposited in the China National Center for Bioinformation (CNCB). The lncRNA-seq, mRNA-seq and circRNA-seq data are derived from the same transcriptome libraries and are deposited under accession number CRA044581; the small RNA (miRNA) sequencing data are deposited under accession number CRA044582.

## Supplementary Materials

The following supporting information can be downloaded at: https://www.mdpi.com/article/10.3390/biology15211994/s1, Figure S1: Survival rate of *A. fasciatus* under different challenge concentrations of *C. freundii* for median lethal dose (LD_50_) determination; Figure S2: Symptoms of diseased *A. fasciatus*. (A) Redness and bleeding on the abdomen and fins. (B) Congested and enlarged liver. (C) Congested and enlarged spleen; Table S1: Primers used for qRT-PCR validation; Table S2: Statistics of 30 Differentially Expressed mRNAs Expression Analysis.
