# Supplementary material for: Integrated Analysis of the lncRNA-miRNA-mRNA ceRNA Network in the Spleen of Acrossocheilus fasciatus Infected with Citrobacter freundii
Source: Biology (Basel). 2026 Nov 4;15(21):1994. doi: 10.3390/biology15211994 (PMC13762065; doi:10.3390/biology15211994)
Supplement: Supplementary file 1 [file biology-15-01994-s001.zip › Supplementary Materials.pdf]

Table S1 Primers used for qRT-PCR validation.

Type	Gene name	Sequence (5’ - 3’)	Product Size (bp)
mRNA	*Serpine1*	F: TGTCCCGCACCAAATAGA	122
		R: GGTCACTCAACACCCCAGA	
	PTGES	F: GCCGATAGACACGGAGGT	202
		R: ATGCGATGCTGTGGAGAA	
	GBP1	F: CGTCTGTCTCCCCTCGTGT	192
		R: GCTTCCCATCTGAGTCCGT	
	C3	F: CCCAGCAAGTCATTCTTCTAT	110
		R: ACACCAAACACCACAAACG	
	NOS2	F: CCTGGCTCAATACCATCAA	112
		R: CCAACTCCCACAAGAATACA	
	A2m	F: CTGGCAGATGCTGATTTTG	113
		R: TGTTTCACAGACCCCACTTC	
LnRNA	*MSTRG.10025.1*	F: CAGGCAGGAATATTTGTAAGTCTA	118
		R: TGAGTGTGGCTGCCTGCCACTCCAC	
	MSTRG.20569.1	F: GTCCATGGCTACAAAATAAGACTGT	127
		R: TCACCGGTCGCCACTACGAAACCAT	
	MSTRG.1229.1	F: CGTGAGCCAAGCGGAGCGCGG	132
		R: GCTCCTCACGGAGAGATGTTATTT	
	MSTRG.13574.1	F: CGAGCTTTTAGGATGACAATTTCCAC	121
		R: TGGATTTCCCGGTGATTATAAGCAA	
	MSTRG.6187.2	F: AATAAAACATATACAGCCGTTATTG	116
		R: TTGCTGAGCAACGACACCCTGCACG	
	MSTRG.7156.2	F: ATGAGTCACCTCATGAGCCAACAGC	122
		R: TGCGCAGCCACCCTGCCATCTCCCAC	
miRNA	*miR-132-y*	F: CGCGTAACAGTCTACAGCCAT	71
		R: AGTGCAGGGTCCGAGGTATT	

	miR-67-y	F: GCGTCACAACCTGCTTGAAT	70
		R: AGTGCAGGGTCCGAGGTATT	
	miR-8-y	F: CGCGCGTAATACTGTCAGGTAAA	73
		R: AGTGCAGGGTCCGAGGTATT	
	miR-212-y	F: CGCGCGTAACAGTCTACAGTCAT	73
		R: AGTGCAGGGTCCGAGGTATT	
	miR-127-x	F: CGCTGAAGCTCAGAGGGCT	69
		R: AGTGCAGGGTCCGAGGTATT	
	miR-381-y	F: GCGTATGCAAGGGCAAGCT	69
		R: AGTGCAGGGTCCGAGGTATT	
Reference gene	*β-actin*	F: CTTCTTGGGTATGGAGTCTTG	218
		R: AGTATTTACGCTCAGGTGGG	
	U6	F: GTGCTCGCCACGGCGGCACA	74
		R: AAAAAGATGGAGCGCTTCAC	

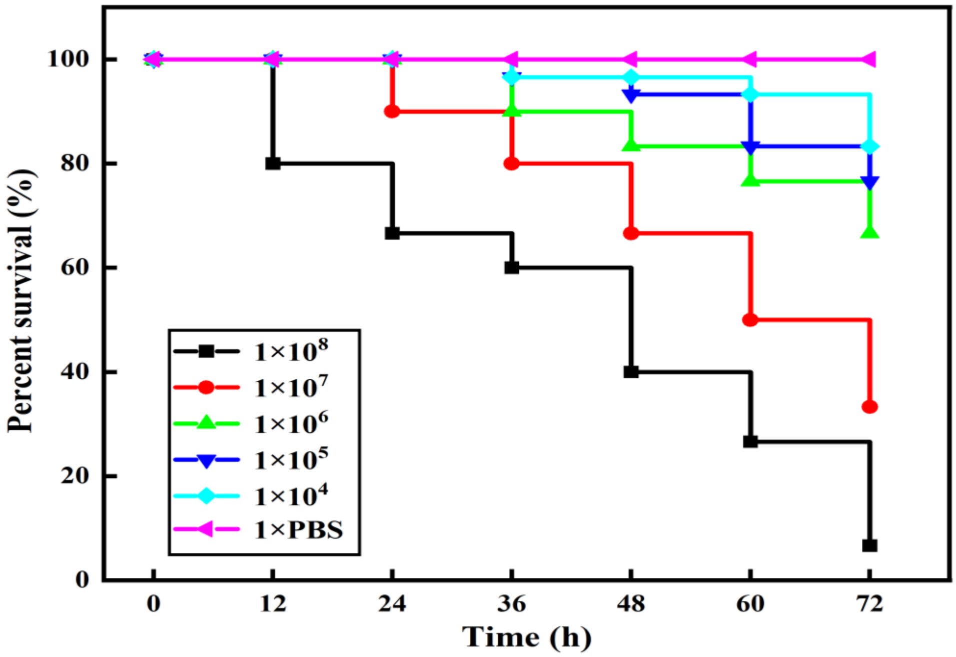

Figure S1. Survival rate of *A. fasciatus* under different challenge concentrations of *C. freundii* for median lethal dose (LD_{50}) determination.

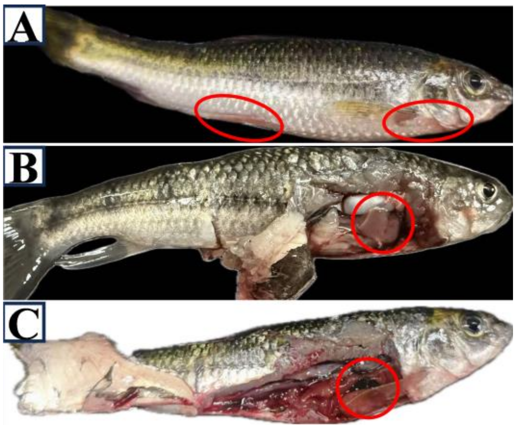

Figure S2. Symptoms of diseased *A. fasciatus*. (A) Redness and bleeding on the abdomen and fins. (B) Congested and enlarged liver. (C) Congested and enlarged spleen.

Table S2 Statistics of 30 Differentially Expressed mRNAs Expression Analysis.

Gene	log2(FC)	*P* Value	FDR	Pathway
Lama3	-5.43	<0.0001	1.61E-06	ECM-receptor interaction
LAMB3	-4.10	<0.0001	5.72496E-05	
Col4a2	-3.91	0.00408	0.027501356	
THBS2	-3.06	<0.0001	1.54588E-08	
VTN	1.45	<0.0001	8.30221E-05	
PTGS2	3.23	0.00002	0.000418	IL-17 signaling pathway
il1b	2.37	<0.0001	3.14163E-06	
ifng1	2.05	0.00001	0.000144231	
CXCL8	1.30	0.00276	0.020264939	
tnf	1.29	0.00825	0.046772454	
CXCL14	-4.27	<0.0001	8.07626E-17	Chemokine signaling pathway
CXCl11.1	2.82	<0.0001	4.72601E-07	
XCR1	2.46	0.00001	0.00023557	
CXCR4	-2.13	<0.0001	1.69338E-31	
CCR7	-2.06	<0.0001	1.93589E-12	
C7	2.92	<0.0001	7.77265E-08	Complement and coagulation cascades
C3	2.15	<0.0001	2.33966E-05	
C5	1.01	0.00158	0.013037431	
C8a	1.51	0.00026	0.003092999	
FGB	2.41	<0.0001	1.62496E-07	
LGMN	-5.46	<0.0001	8.3387E-6	Antigen processing and presentation
CTSL	-5.06	0.00012	0.001573151	
TAP1	1.56	<0.0001	4.11603E-14	

H2-Ab1	-2.88	<0.0001	1.19202E-12	
H2-Aa	-2.11	0.00061	0.006099952	
Tlr2	-1.16	<0.0001	3.93019E-12	Neutrophil extracellular trap formation
AQP9	-2.55	0.00269	0.019885869	
caspa	2.28	<0.0001	7.55197E-06	
c3ar1	-1.60	0.00002	0.000375346	
MARCO	-1.93	<0.0001	9.14803E-15	Phagosome
